# Validation of cross-cultural child mental health and psychosocial research instruments: adapting the Depression Self-Rating Scale and Child PTSD Symptom Scale in Nepal

**DOI:** 10.1186/1471-244X-11-127

**Published:** 2011-08-04

**Authors:** Brandon A Kohrt, Mark JD Jordans, Wietse A Tol, Nagendra P Luitel, Sujen M Maharjan, Nawaraj Upadhaya

**Affiliations:** 1 Transcultural Psychosocial Organization (TPO) Nepal, Baluwatar, Kathmandu, Nepal; 2 HealthNetTPO, Amsterdam, The Netherlands; 3 Global Health Initiative, Yale University, New Haven, USA; 4 Central Department of Psychology, Tribhuvan University, Kirtipur, Nepal; 5 Dept of Anthropology, University of Amsterdam, Amsterdam, The Netherlands

## Abstract

**Background:**

The lack of culturally adapted and validated instruments for child mental health and psychosocial support in low and middle-income countries is a barrier to assessing prevalence of mental health problems, evaluating interventions, and determining program cost-effectiveness. Alternative procedures are needed to validate instruments in these settings.

**Methods:**

Six criteria are proposed to evaluate cross-cultural validity of child mental health instruments: (i) purpose of instrument, (ii) construct measured, (iii) contents of construct, (iv) local idioms employed, (v) structure of response sets, and (vi) comparison with other measurable phenomena. These criteria are applied to transcultural translation and alternative validation for the Depression Self-Rating Scale (DSRS) and Child PTSD Symptom Scale (CPSS) in Nepal, which recently suffered a decade of war including conscription of child soldiers and widespread displacement of youth. Transcultural translation was conducted with Nepali mental health professionals and six focus groups with children (n = 64) aged 11-15 years old. Because of the lack of child mental health professionals in Nepal, a psychosocial counselor performed an alternative validation procedure using psychosocial functioning as a criterion for intervention. The validation sample was 162 children (11-14 years old). The Kiddie-Schedule for Affective Disorders and Schizophrenia (K-SADS) and Global Assessment of Psychosocial Disability (GAPD) were used to derive indication for treatment as the external criterion.

**Results:**

The instruments displayed moderate to good psychometric properties: DSRS (area under the curve (AUC) = 0.82, sensitivity = 0.71, specificity = 0.81, cutoff score ≥ 14); CPSS (AUC = 0.77, sensitivity = 0.68, specificity = 0.73, cutoff score ≥ 20). The DSRS items with significant discriminant validity were "having energy to complete daily activities" (DSRS.7), "feeling that life is not worth living" (DSRS.10), and "feeling lonely" (DSRS.15). The CPSS items with significant discriminant validity were nightmares (CPSS.2), flashbacks (CPSS.3), traumatic amnesia (CPSS.8), feelings of a foreshortened future (CPSS.12), and easily irritated at small matters (CPSS.14).

**Conclusions:**

Transcultural translation and alternative validation feasibly can be performed in low clinical resource settings through task-shifting the validation process to trained mental health paraprofessionals using structured interviews. This process is helpful to evaluate cost-effectiveness of psychosocial interventions.

## Background

The dearth of mental health and psychosocial support (MHPS) research in low- and middle-income countries (LAMIC) is a barrier to providing evidence-based care to children and youth. Of the more than two billion children in the world, the majority lives in LAMIC. Half of these children live in poverty, two-thirds are affected by armed conflict, and a third is underweight or stunted [1], yet these vulnerable populations comprise only five percent of published child mental health literature [2].

The adaptation and validation of instruments to assess MHPS for use in LAMIC is crucial to eliminate this gap in child global mental health research and service provision [2,3]. Hospital records, structured diagnostic interviews in clinical settings, and school-based psychological testing have been used extensively in high-income countries to assess both prevalence of childhood mental illness and the response of children to intervention. However, hospital records and clinical diagnostic interviews are typically lacking in LAMIC because of the absence of child psychiatric services. Therefore, the use of instruments that can be administered to teachers, parents, and children are a helpful alternative to gain information until clinical services are more established. Without validated instruments, resources are easily misallocated through either providing care to children not requiring services or depriving care to children who desperately need it. Furthermore, the psychometric properties of validated instruments can be used to estimate costs of service provision or deprivation.

However, instruments developed and validated with children in high income countries with Western cultural settings cannot simply be translated with the expectation they will have the same psychometric properties in other cultural contexts. Cutoff scores established with Western child populations are not necessarily comparable in other settings and may lead to misclassification and distortion of prevalence rates [4,5]. Moreover, the instruments may not capture the constructs they are intended to measure in other cultural contexts where the meaning, clustering, and experience of symptoms often differs [6-8]. In humanitarian emergencies in particular, data from unvalidated instruments can be worse than no data at all because it may lead to inappropriate and potentially harmful intervention [9,10]. Without the use of validated screening instruments, psychosocial interventions unintentionally may divert resources from the children most in need of mental health services. Without validated screening instruments, it is not possible to evaluate the effectiveness of an intervention, thus risking potential failure of programs to improve children's lives and simultaneously wasting scarce human and economic resources.

Questionnaires, therefore, need to be validated in any new socio-cultural setting. However, the process and interpretation of validation procedures are not straight forward, especially in cross-cultural context. The definition, determination, terminology, and interpretation of *validity *vary by discipline, available resources, and type of problem studied. Cross-cultural validation techniques have been developed for adults [11-15], and there are examples for children and adolescents, as well [16,17]. However, there has not been agreement on a single validation method most appropriate for global mental health research with adults or children. The sole consensus has been that only translating and back-translating falls short of producing valid tools [4,18]. With increased emphasis on *task-shifting *in mental health care and training [19], it may be possible to have experienced non-psychiatrists conduct validation evaluations using structured interviews. Task-shifting refers to the process of having midlevel professionals, such as nurses and physician's assistants, and paraprofessionals, such as community psychosocial workers, take on responsibilities assumed by psychiatrists and psychologists in high-income settings. Task-shifting is warranted because of the dearth of high-level professionals in LAMIC. Ultimately, an array of validation approaches may prove most useful because the variation in types and intended uses of measures in global mental health and psychosocial research.

Our goal, therefore, is to discuss how to judge and interpret validity of child MHPS instruments across settings and cultures rather than advocate a one-size-fits-all approach to conducting validation. We propose *six evaluation questions *to be asked when validating an instrument or selecting among validated instrument for use in cross-cultural MHPS research. We present these questions in a manner usable by practitioners in non-governmental, humanitarian, and other development organizations, which are the dominant arenas for monitoring and evaluation of child MHPS interventions. We employ the six questions to analyze transcultural translation and validation of instruments with conflict-affected youth in Nepal. We conclude with a discussion of the economic implications of using validated MHPS instruments.

### Six questions to appraise cross-cultural validity for child mental health and psychosocial measures

#### 1. What is the purpose of the instrument?

Validity is not an inherent property of an instrument. Validity varies by setting and population. Instruments valid for one study may not be valid for other purposes even in the same setting. For example, PTSD measures validated for prevalence studies do not have demonstrated utility in treatment planning even in Western settings [20]. In global MHPS research with children, there are myriad purposes for using instruments: screening children exposed to war, natural disasters, or chronic poverty [16,17], estimating prevalence of disorders [21], measuring treatment response to MHPS interventions [22-25], and exploring biological processes of child mental health cross-culturally [26].

To clarify the purpose of an instrument, knowing the context is crucial. The context for our study was a decade-long conflict between the Government of Nepal and the Communist Party of Nepal-Maoists from 1996-2006. After the war's conclusion, international organizations such as UNICEF, Save the Children, Plan, and the International Rescue Committee channeled funding to Nepali nongovernmental organizations (NGOs) to provide psychosocial care for children affected by armed conflict including both child soldiers and civilian children. In this context, we worked with Transcultural Psychosocial Organization (TPO) Nepal, an NGO that was involved in a number of trainings, interventions, and research projects to support war affected youth. We chose to undertake this transcultural translation and validation study for the purpose of developing and adapting instruments that could be used to screen children for enrollment in NGO psychosocial interventions, compare differences in need for intervention between groups such as child soldiers versus war affected civilian children [27], and measure the effectiveness of interventions to enhance resilience and reduce psychosocial disability related to depression, PTSD, and other forms of MHPS problems [28]. Without validated instruments, it would not have been possible to assess and interpret the impact of these interventions.

#### 2. What is the construct to be measured?

This second question addresses how well the category captures the lived experience of a presumed category of distress, a concept known as *construct validity*. Types of constructs to be measured can be divided into three categories: local constructs, Western psychiatric constructs, and cross-cultural constructs. Local constructs, also referred to as idioms of distress or culture-bound syndromes, have the advantage of being salient to the target community so that screening and intervention is consistent with local priorities [29]. Alternatively, researchers may be more interested in looking for the manifestation of Western psychiatric constructs such as PTSD or depression [21] regardless of whether it is meaningful, significant, or associated with distress as recognized by the local group. While such work has little salience for participating communities at the time of the study, such studies putatively garner international policy and financial attention [30]. Cross-cultural constructs are assumed to have commonalities across cultural groups and settings. Cross-cultural studies explore differences in symptoms, risk and protective factors, social interpretation, stigma, and treatment response while maintaining the assumption that there are common processes at work between cultures [31-33].

Cross-cultural constructs differ from pure Western psychiatric constructs in that the former assume there is a shared meaning across cultures. While some epidemiologists would primarily focus on the presence or absence of a symptom across cultural groups, a cross-cultural investigator would be concerned about whether or not there were shared meanings across cultural groups. The latter process requires substantial ethnography and other qualitative research. Bolton and colleagues have developed a validation approach that uses rapid ethnographic measures to pronounce cross-cultural applicability when concordance between Western measures and local idioms is demonstrated [15]. However, work such as this is the exception rather than the rule for child MHPS programs in LAMIC. Our concern is that often Western psychiatric constructs are uncritically and inflexibly applied in humanitarian emergencies through simple translation of English-language scales without taking time to understand what both the individual items and the broader construct mean in a different cultural setting. Western psychiatric constructs may have utility or even possibly universality. However, significant attention to each item, symptom, and category of experience is needed before cross-cultural relevance can be accepted. Beginning with Western psychiatric concepts as a starting point has merit as long as this is *only *a starting point and researchers are open to reexamining the utility and relevance through in-depth ethnographic, participatory, and experience-near research.

Once the purpose has been clearly defined, it is easier to determine the appropriate construct. For example, a prevalence study will typically require selecting a Western psychiatric construct then performing validation against a clinical diagnosis. In contrast, a screening or treatment response study could use a local or cross-cultural construct and the external criterion for validation could be the risk of exposure to a traumatic event [14] or functional impairment [34]. For our study, we chose the psychiatric categories of depression and PTSD, with the view that they can operate as cross-cultural constructs salient for Nepali populations and be interpreted easily by international academic and humanitarian donor audiences.

Ethnographic research in Nepal revealed that there are not concepts directly synonymous with clinical depression and PTSD within Nepali cultures. However, aspects of these phenomena were observable, associated with distress, and had salient terminology to capture specific elements of the disorders [35,36]. The local categories of distress were relevant to children's experiences of war [37-39]. Therefore, our framework, which was grounded in over a decade of ethnography in Nepal, assumed sufficient shared cross-cultural experience to select depression and PTSD questionnaires for adaptation in the Nepali post-conflict setting.

#### 3. What are the contents of the construct?

After determining whether the study is going to assess a local construct, Western psychiatric construct, or cross-culturally salient construct, the constituent elements of the category need to be determined. These may be social relations, internal states, behaviors, exposures, personal characteristics, or other symptoms. If a local construct is being investigated, the constituent elements are typically identified through qualitative methods, ethnography, and specific tasks such as freelists and pile sorts [18,40]. At the crudest level, content differences need to be adjusted for the setting. Western instruments may refer to behaviors and experiences that are not applicable in other locales. For example, "stands quietly when in line", an item commonly used to assess ADHD among schoolchildren in high-income countries, was not an applicable item for ADHD in Nepal because it was not a common task for some children [18]. The technical terms for this question are *content validity *and *content equivalence*: "the content of each item of the instrument is relevant to the phenomena of each culture being studied," [12].

#### 4. What are the idioms used to identify psychological symptoms and behaviors?

Specific language for items should be selected carefully. Idioms related to behavior and inner states are culture-specific and rarely translatable in a literal manner. The term 'ashamed' (*vergüenza) *has negative connotations in Spanish but 'uncomfortable' (*incómodo*) could be used [13]. Similarly, direct translation of the benign term 'adventure' from English into Spanish changed connotation to sexual escapades [12]. When idioms in different cultures reflect similar underlying phenomena this is *semantic equivalence*, "the meaning of each item is the same in each culture after translation into the language and idiom (written or oral) of each culture," [12].

#### 5. How should questions and responses be structured?

The next cultural issue to consider after determining the appropriate idioms and phrases is how best to ask a question. Instruments range from using true and false declarative statements to interrogatives. Response sets may be categorical 'yes/no' or may include severity levels on a Likert scale, e.g. 'rarely' to 'often'. Alternatively, response sets may be illustrations. In Afghanistan, water glasses filled to different levels represented different response categories [14]. In Uganda, pictures with women carrying different loads on their heads symbolized severity levels [41]. *Technical equivalence *is achieved when, "the method of assessment... is comparable in each culture with respect to the data that it yields," [12]. Technical equivalence implies that response sets capture similar declinations of severity across cultural groups. In Nepal, previous research identified problems using Likert scales and categorization of questionnaire response sets [18]. Therefore, our research also piloted different approaches to quantifying symptom severity, such as through the use of locally developed illustrations.

#### 6. What does a score on the instrument mean?

When a measure is classified as 'valid', this typically refers to having undergone a comparison with a clinical diagnosis. The term 'gold standard' validation is often invoked when the external criterion is clinician-rated structured interview. This is known as *diagnostic validity *[42], which is one form of *criterion validity *[7]. Therefore, the instrument score is a proxy for that diagnosis. However, other proxies can be external criterion such as scores on other validated instruments, level of known risk or protective factors, biological risk factors, physiological outcomes, genetic measurements, or future outcomes such as school performance, substance abuse, or violent behavior as adults. The intended purpose of the instrument should dictate the type of comparison. Clinical 'gold standard' interviews are not the ideal comparisons in all instances, and 'gold standard' validation may not be feasible because of the lack of child mental health specialists in LAMIC. The Afghan Symptom Checklist validation compared the instrument score with level of exposure to war trauma [14]. These approaches are useful to establish *concurrent validity*, i.e. a significant relationship between the instrument scores and another measure [42]. Ultimately, for prevalence studies, diagnostic validation is crucial. The misapplication of instruments that have not undergone diagnostic validation to make prevalence claims is one of the most common errors in global mental health research.

For our research in post-conflict Nepal, we needed instruments that could identify children with significant levels of MHPS-related disability, provide prevalence estimates of depression and PTSD for academic and donor audiences, and quantify treatment response. Therefore, the instruments needed to assess cross-cultural constructs comprising locally meaningful phenomena. We required external validation criteria that included both a measure of disability and a structured assessment of depression and PTSD. Because of the paucity of child mental health specialists, we implemented an alternative process of task-shifting using a trained psychosocial counselor equipped with a structured clinical interview and ordinal disability ranking tool.

## Methods

### Setting

Nepal, a landlocked South Asian country, endured an eleven-year war that ended in 2006 and claimed over 14,000 lives [43]. Poverty and discrimination are major influences on child wellbeing that predate the conflict and continue to exist since its resolution [44] Children were affected through war exposures ranging from displacement to bombings, and thousands of persons under 18 years of age were conscripted into armed groups [27,39]. Nepali is the national language and used in the majority of educational institutions. Hinduism and Buddhism are the dominant religions in the country.

### Instruments

The Depression Self Rating Scale (DSRS) is an 18-item self-report measure for children [45], which has been used in a range of cross-cultural contexts [46-48]. This instrument records symptoms over the past week. Items are presented as statements, e.g. "I sleep very well." Responses are a 0 'mostly', 1 'sometimes', 2 'never'.

The Child PTSD Symptom Scale (CPSS) was developed as a child-version of the Posttraumatic Diagnostic Scale [49,50]. The CPSS has 17 items that correspond to PTSD diagnostic criteria in the *Diagnostic and Statistical Manual of Mental Disorders *(DSM-IV) [51]. Part 2 of the instrument includes 6 items related to impairment in functioning. Items are provided as statements, e.g. "having bad dreams or nightmares." Children score these items on a 0-4 scale based on frequency over the past week: 0 'not at all or only one time', 1 'once a week or less, once in a while'; 2 '2-4 times a week, half the time,' 3 '5 or more times a week/almost always'. Part 2 of the instrument records impairment in different areas of life. Although this second section was translated, it is not included in the analyses here because a separate independent Child Function Impairment instrument was developed for the child research in Nepal.

### Transcultural translation

According to an established transcultural translation procedure [11], four criteria are evaluated at each qualitative research step: comprehensibility, acceptability, relevance, and completeness. *Comprehensibility *is a measure of semantic equivalence. Comprehensibility relates to Question #4 pertaining to using appropriate idioms. If an item is deemed to be comprehensible by a focus group or individual, it is assumed to be understandable by a general audience in the specific cultural setting.

*Acceptability and response set issues *reflect technical equivalence in how data are collected across cultures. Question #5 concerns the culturally salient approach to ask questions and score responses. If an item is deemed to have an acceptable response set, it suggests that respondents will rate items similarly to the original intention of the instrument.

*Relevance *of items demonstrates content equivalence. Whereas comprehensibility captures whether an item is understood though local idioms, relevance is a measure of whether the item has locally significant meaning. For example, even though children may understand an item related to "watching television" or "playing video games," the item may not be relevant in some LAMIC settings where only elite children have access to these leisure activities. Relevance information can be used to answer Question #3 regarding the contents of the construct.

*Completeness *combines semantic, criterion, and conceptual equivalence, thus capturing whether a question relates to the same concepts and ideas as the original item. Completeness accounts for cultural norms in relation to markers of psychopathology. For example, even though decreased sexual interest may be a comprehensible item (people understand the terms) and relevant (sexual relations occur in the majority of the world's cultures), it may not be a marker of depression in a culture where it is not acceptable for women to endorse interest in sex. Both depressed and non-depressed women would be equally likely to endorse low sexual interest in that culture. The criterion of completeness can thus be employed to answer Question #2 regarding the construct to be measured; for example, does the item reflect the experience of depression or PTSD.

In the first step of the our transcultural translation, a team of three native Nepali speakers trained in English and one native English speaker trained in Nepali all of whom had mental health expertise evaluated each DSRS and CPSS item according to the four criteria described above. Second, a Nepali psychiatrist and a Nepali psychologist, both of whom had years of clinical experience in Nepal, independently reviewed each item and commented on the four criteria. Modifications were made to the items based on their recommendations.

The third step comprised focus group discussions with Nepali children whose age, ethnic, and residential demographics were comparable to the children who would later participate in quantitative studies. Six focus groups were conducted, three with boys (n = 32) and three with girls (n = 32) aged eleven to fourteen years old. The instruments were modified according to the children's recommendations. Children also evaluated three pictographic response scales drawn by a Nepali artist: water glasses, an abacus, and a *dhoko*-basket scale.

For the fourth step, a bilingual Nepali-English speaker who was blinded to the original instruments reviewed the Nepali items that had been modified by both the mental health professionals and children. The bilingual speaker back-translated these into English for comparison with the original. The original English and final English back-translation were reviewed by the study team to address any remaining concerns related to completeness of the translations. These four steps were conducted during October-December 2006. See additional files 1 and 2 for the final Nepali translations and final English back-translations of the DSRS and CPSS.

### Validation

The Kiddie-Schedule for Affective Disorders and Schizophrenia (K-SADS) [52] and Global Assessment of Psychosocial Disability (GAPD) [53] were selected as structured instruments that could be used in a clinical interview to assess depression, PTSD, and level of psychosocial disability. The K-SADS is a child version of the adult Schedule for Affective Disorders and Schizophrenia [54]. It is a semi-structured diagnostic interview to be administered by trained research and clinical personnel. The K-SADS allows trained interviewers to score children on DSM-IV diagnoses. For this study, a psychosocial counselor was trained to use specific modules of the K-SADS in order to identify depression, PTSD, or other psychosocial difficulties. During the training period, the psychosocial counselor was supervised by an expatriate psychologist and psychiatrist. After training, the psychosocial counselor categorically scored the children as meeting or not meeting DSM-IV criteria for major depressive disorder and PTSD.

The GAPD is derived from Axis VI on the multiaxial presentation of the *International Classification of Mental and Behavioural Disorders *(ICD-10) [55], and is comparable to Axis V on the DSM-IV multiaxial formulation [51]. The GAPD score is based on functioning in domains of personal motivation, school performance, family relations, peer relations, and occupational functioning. Impairment (high scores on the GAPD) is scored only when disability can be attributed to mental health problems. The GAPD has been adapted for use with children [53]. Trained clinicians score children from zero (no impairment) to eight (extreme impairment). In this study, the rater was a psychosocial counselor trained on assessments using the GAPD. The psychosocial counselor's assessment was compared with the expatriate psychologist's and psychiatrist's assessments of children until sufficient concordance of ratings could be achieved.

A psychosocial counselor was chosen to perform the GAPD and K-SADS ratings because there were no Nepali certified specialists in child psychology or psychiatry at the time of the study. A psychosocial counselor was selected because this is the most common level of MHPS provider for children in Nepal [56]. These counselors have the greatest experience with children specifically in the area of mental health so we hoped someone from this discipline would have the best ability to judge which children were in need of MHPS intervention. We recruited a psychosocial counselor with six months of classroom and clinical training and two years of experience working with children with emotional-behavioral problems. The selected psychosocial counselor received three weeks of training on the K-SADS and the GAPD, which included rating children and reviewing these rating with the two internationally trained mental health professionals, as described above. The psychosocial counselor rated children in four areas: depression caseness, PTSD caseness, other psychosocial caseness, and GAPD score.

### Participants

We randomly selected one school for participant recruitment using a list of all accessible schools in the targeted district. We chose this district because it was within the catchment region of the psychosocial intervention to be evaluated. Permission was obtained from the principal. Children were randomly selected from school rosters for 6^th ^and 7^th ^grade with the age range of 11-14 years old. These selected children were enrolled in the study if parental consent was provided. No child-parent dyads refused participation. The final sample was 162 school children. Children were interviewed by research assistants trained in administration of the CPSS and DSRS. Children were then interviewed by the psychosocial counselor who was blinded to the results of the CPSS and DSRS. The validation component was conducted during May-July 2007. The group was bifurcated into an indication-to-treat group versus no-indication. Criteria for the indication-to-treat group were having a GAPD score greater than four and caseness determined by the K-SADS, with both determinations made by the psychosocial counselor.

### Statistical analyses

Table 1 lists the statistical concepts related to instrument validation. Statistical analyses were done with SPSS 16.0 [57], and included paired t-tests to compare the averages of instrument total scores between indicated-to-treat and non-indicated groups, as well as receiver operator characteristics (ROC) curves and area under the curve (AUC). Diagnostic sensitivity and specificity, positive predictive value, negative predictive value, and reliability were calculated. Individual items also were compared between the two groups. Bonferonni-type corrections were made for these analyses because of the multiple tests conducted; statistical significance was multiplied by the number of tests, 18 for DSRS and 17 for CPSS. Otherwise, a p-value of 0.05 was used to determine significance.

**Table 1:** Statistical terminology for validated instruments and interpretation of child mental health and psychosocial support (MHPS) research in Low and Middle Income Countries (LAMIC)

	Concept	Calculation	Application to child MHPS research in LAMIC
**Area under the curve (AUC)**	The probability that the instrument will yield a higher score for a randomly chosen individual with the target condition than for a randomly chosen individual without the condition	Area under the graph with sensitivity on the *Y *axis by one minus specificity on the *X *axis	The ideal instrument for screening and/or evaluation of an intervention for children in LAMIC will have a high AUC (close to 1.0). The closer to 0.5 the AUC, the less utility of the screening instrument and the less cost-effectiveness of screening

**Cutoff score**	The score on the instrument chosen to differentiate cases from non-cases; may be chosen to maximize specificity, sensitivity, or both	Chosen by researcher based on ROC curve	Based on the type of intervention program, a higher or lower cutoff score could be chosen to prioritize sensitivity or specificity

**Sensitivity**	The ability of an instrument, at a selected cutoff score, to identify persons with a target condition. At a sensitivity of 1.0, all persons with the condition are identified, and there are no false negatives		Instruments with high sensitivity are ideal to screen children when trying to identify the majority of children in distress needing intervention. At high sensitivity, few children with a condition will be mistakenly deprived of the intervention

**Specificity**	The ability of an instrument to include persons who do not have the target condition below the cutoff score. At a specificity of 1.0, no persons without a target condition score above the cutoff		Instruments with high specificity minimize the number of children who are incorrectly identified with a high score, but who do not have the target condition. Specificity is a concern when there are negative consequences to being inappropriately included in an intervention, such as stigma or high expense

**Positive predictive value (PPV)**	The proportion of persons with scores above cutoff who are correctly classified as having the target condition compared to all persons who score above the cutoff		PPV produces more accurate cost estimates of improperly *including *participants than specificity alone because of accounting for prevalence of a condition in the target population

**Negative predictive value (NPV)**	The proportion of persons who score below the selected cutoff who do not have the target condition compared to all persons below the cutoff		NPV is used to determine the proportion improperly *excluded *from an intervention, taking prevalence into account. NPV helps to estimate the cost of not including a proportion of children in an intervention

**Reliability (Cronbach's alpha)**	A measures of internal consistency based on the degree of inter-correlation among all items on a scale		Reliability is important for newly developed measures or adapted measures in LAMIC to help identify items that may not be culturally or contextually relevant, such as stomachaches in Nepal

Abbreviations: Receiver operating characteristic (ROC) curve is the graphical plot of sensitivity and 1-specificity. True Positives (TP) are persons who score above the selected cutoff and have the target condition; True Negatives (TN) are persons who score below the selected cutoff and do not have the target condition; False Positives (FP) are persons who score above the selected condition but do not have the target condition; False Negatives (FN) are persons who score below the selected cutoff but do have the target condition. For the Cronbach's alpha calculation, *K *is the number of instrument items,  is the average of all covariances between the components, and  is the average variance.

### Informed consent and ethical approval

All participants and their caregivers participated in an informed consent process. Children provided assent, and their caregivers provided consent. Children and families received no monetary compensation for participation. Children received snacks while participating in focus groups or individual interviews. The transcultural translation and validation research protocol was approved by Emory University Institutional Review Board, Atlanta, USA, and by the Nepal Health Research Council, Kathmandu, Nepal.

## Results

### Contents of the construct (*content equivalence*)

The goal of content equivalence was to determine if items were relevant to the overall constructs of depression and psychological trauma. Every DSRS item was endorsed by at least two children from each focus group as having a connection to *dukkha *(sadness), with the exception of two items. No child endorsed an association of "stomachaches" (DSRS.6) or "enjoying food" (DSRS.8) with *dukkha*. One child referred to these as "foolish questions" because "anyone can get a stomachache, whether you are sad or happy." Another child explained, "Stomachaches are easy. Everyone gets them."

On the CPSS, the item of avoiding places, people, and activities that recall the traumatic event (CPSS.7), raised relevance concerns in the context of a war-affected setting. In every focus group, at least two children said avoiding places and people involved in the conflict was a natural response. At least one child per group said children should not visit places where accidents, traumas, or other violence occurred because ghosts and spirits of the deceased haunt these places. One mental health worker also reported that avoidance was not a relevant item to identify pathology because "Children see war every day. If they did not avoid dangerous places, they would not be alive." No child in any focus group reported that traumatic amnesia (CPSS.9), i.e. not remembering specific elements of a traumatic exposure, was related to distress. At least one child per focus group expressed that "not remembering" was a good response seen among children without distress.

### Terminology and idioms for items (*semantic equivalence*)

When examining semantic equivalence, no child in any focus group reported difficulty with the Nepali terminology used to inquire about poor sleep, crying, bad dreams, and being easily startled. For some items, we used focus group findings to change terminology (See Additional file 2 for details on specific changes).

Other items required the addition of examples and qualifiers. In the DSRS, "looking forward to things" (DSRS.1) required an example because at least two children identified this item as unclear. Two children in different focus groups independently suggested adding "visiting one's maternal uncle," to DSRS.1. This was affirmed by other children in the focus groups as an event which children anticipate positively. Bilingual mental health workers stated that there was not a direct Nepali equivalent for the item "stick up for myself" (DSRS.9). Therefore, we changed the item to "speaking up when one suffers or witnesses an injustice," as suggested by one child in a focus group. The item "I am easily cheered up" (DSRS.16) required a qualifier of the amount of time it takes to feel happy after being sad. Children in three focus groups reported that one should be cheered up within 5-6 minutes.

In the CPSS, the phrase "feeling guilty" in relation to a traumatic event was removed from the question of distress upon re-exposure (CPSS.4) because at least one child in every focus group said that asking about guilt implied the child should feel guilty. At least one child in every focus group interpreted the item "feeling close to people around you," (CPSS.10) literally as physical distance. Therefore, in order to capture the intended meaning, the clause "close in your heart-mind" was added to evoke emotional closeness, which all children reported was understandable. Similarly, the item related to irritability and fits of anger (CPSS.14) did not appear connected to trauma for the children because two or more children per focus group did not understand the terminology selected for irritability. Therefore, the clause "get angry in small matters" was added. No child reported difficulty understanding this term.

### Structure of questions and responses (*technical equivalence*)

The structure and response set (technical equivalence) of the DSRS and CPSS posed challenges for the children in focus groups. Because the DSRS and CPSS items are structured as declarative statements, the children understood the statements as demonstrative about them, i.e. that the interviewer was stating a fact about the children. At least one child per focus group said that items worded in declarative fashion, e.g. "I feel very lonely", implied that a child should say 'yes'. In contrast, all children commenting across the six focus groups said it was easy to respond to interrogative versions of the items, such as "How often do you feel lonely?" Therefore, we changed all items question form. Children in all focus groups reported that this corresponded with non-coercive styles of conversation.

A second challenge was ordering of the answer set for the DSRS, which ranged from 0 for 'mostly' to 2 for 'never'. At least one child in every focus group reported that presenting response categories starting with 'mostly' and ending with 'never' was backwards and confusing. Every child who responded to the question about order of items stated that is was easier to answer with response sets in the order of 'never' to 'sometimes' to 'mostly' rather than vice versa. We therefore changed the ordering and adjusted the numeric values to correspond with the DSRS standard scoring. No child reported difficulty with CPSS response set that was ordered from 'never' to 'often'.

**Figure 1:**
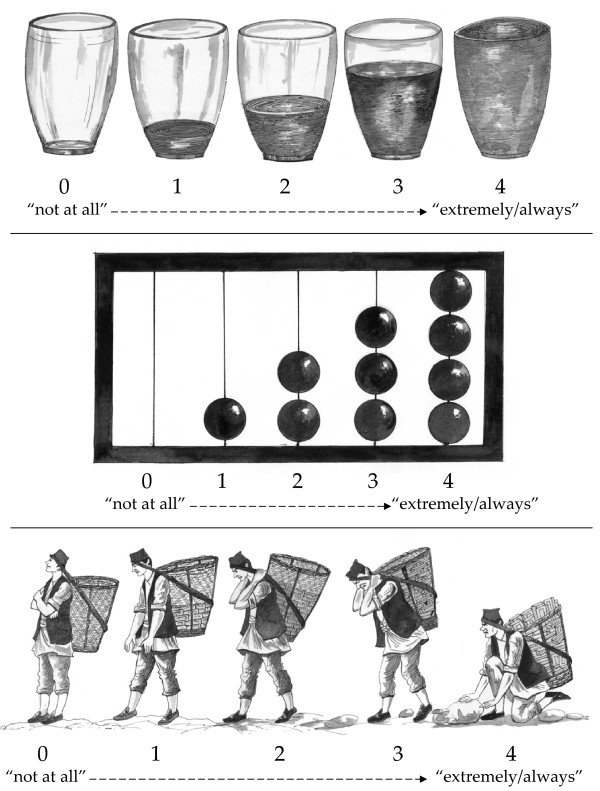
**Picture-based response sets: water glasses, abacus, and dhoko-basket scales**. Children in focus groups reviewed these three drawing series to determine appropriate pictorial response sets to maintain technical equivalence. The water glasses and abacus scales were generally understood. The *dhoko*-basket scale was not used because children consistently identified option '0' (empty basket) as 'sad' or 'lazy' because the boy had no bricks in his basket and would therefore earn no money compared with '4' (full basket), which was associated with happiness because of high earning potential with a large number of bricks.

We elicited children's views on the three pictographic scales: water, abacus, and *dhoko *(basket) scale (see Figure 1). The *dhoko*-basket scale was developed as a modification of Bolton and Tang's non-verbal response card depicting persons carrying bags with different gradations of weight [41]. The *dhoko*-basket scale ranged from a man with no bricks in his basket standing upright to the other extreme of a man with a *dhoko*-basket full of bricks. The last man is perspiring and straining under the heavy weight. We explained to children that the *dhoko*-basket represented their *man *(heart-mind) and the bricks represented an emotion such as anger, sadness, or fear. They were told to describe how much their heart-mind was full of a specific emotion by choosing a *dhoko*-basket with a specific quantity of bricks. We expected children to associate an empty *dhoko*-basket with the positive condition of being symptom free and to associate a full *dhoko*-basket with an undesirable condition of heavy symptom burden.

The responses by children regarding the *dhoko*-basket scale ran counter to our expectations. In every focus group, two or more children associated a full *dhoko*-basket with lack of sadness and an empty *dhoko*-basket with extreme sadness. After encountering this multiple times, a boy in one focus group explained,

"Number 4 [the man with a *dhoko*-basket full of bricks] is always happy in this picture because he has the most bricks. The more bricks, the more money you are going to make. The man with the empty basket is lazy and doesn't have even one brick. He will not make any money and then he will become very sad."

The boy who provided this response and others in his focus group interpreted the bricks in terms of their financial implications rather than level of physical exertion. Children viewed a full *dhoko*-basket as exemplifying an individual with work whereas the empty *dhoko*-basket represented an individual with no load and thus no employment. While no children in other groups provided this exact explanation, when presented with the interpretation of lazy and sad as an empty basket and happy as a full load of bricks, every responding child said this interpretation was more plausible than the converse. Therefore, the *dhoko*-basket scale was discarded for the quantitative section of the study. Of the three picture scales, the water glasses were the easiest to translate into response sets. Three children encountered difficulty in abstracting the abacus bead levels to symptom severity level.

### Validation (*criterion/diagnostic validity*)

Once the instrument items were transculturally translated, they were piloted with 162 children (see Table 2 for demographics). The 162 children participated in the structured GAPD and K-SADS interview with the trained psychosocial counselor in addition to completing the DSRS and CPSS with other trained research assistants. The psychosocial counselor identified 28 children (17%) in need of psychosocial intervention using a GAPD score greater than four in the structured interview as the criterion. Children whom the psychosocial counselor scored above four on psychosocial disability had higher mean scores on the DSRS and CPSS (see Table 3 for means, area under the curve (AUC), cutoff score, sensitivity, and specificity).

**Table 2:** Socio-demographic characteristics of validation sample

	No-Indication to treat (n = 134)	Indication to treat (n = 28)	Total (n = 162)
*Gender*			
Boys	40 (29.9))	12 (42.9)	52 (32.1)
Girls	94 (70.1)	16 (57.1)	110 (67.9)

*Age*			
11	7 (5.2)	2 (7.1)	9 (5.6)
12	28 (20.9)	3 (10.7)	31 (19.1)
13	35 (26.1)	9 (32.1)	44 (27.2)
14	64 (47.8)	14 (50.0)	78 (48.1)

*Level of education*			
Grade six	18 (13.4)	6 (21.4)	24 (14.8)
Grade seven	116 (86.6)	22 (78.6)	138 (85.2)

*Caste/Ethnicity*			
Bahun/Chhetri	75 (56.0)	18 (64.3)	93 (57.4)
Dalit (Nepali, BK)	15 (11.2)	5 (17.9)	20 (12.3)
Tharu	34 (25.4)	5 (17.9)	39 (24.1)
Others (Magar, Newar & Lodcha)	10 (7.5)	-	10 (6.2)

*Religion*			
Hindu	132 (98.5)	28 (100.0)	160 (98.8)
Buddhist	2 (1.5)	-	2 (1.2)

**Table 3:** Validation psychometric properties

		DSRS (18 items)	CPSS (17 items)
*No indication to treat group (n = 134)*	Mean (SD)	11.0 (3.2)	16.5 (5.8)
*Indication to treat group (n = 28)*	Mean (SD)	15.6 (4.1)	22.6 (6.4)

*Group Differences*	T-test	6.52	5.00
	p-value	<. 001	<. 001

*Psychometrics*	AUC	0.82	0.77
	Cutoff score	≥ 14	≥ 20
	Sensitivity	0.71	0.68
	Specificity	0.81	0.73
	Positive Predictive Value	0.36	0.35
	Negative Predictive Value	0.95	0.92
	Reliability (Cronbach's alpha)	0.67	0.86
	Test-Retest Reliability	0.80	0.85

For the DSRS, with a cutoff score of 14 or greater indicating need for treatment, 20 children (12.3% of the total) were correctly classified as having psychosocial disability according to the GAPD (true positives); 108 children (66.7%) were correctly classified as not having psychosocial disability (true negatives). However, 26 (16.0%) were incorrectly classified according to the DSRS as requiring intervention, but the psychosocial counselor did not classify these children as psychosocially disabled according the GAPD rating (false positives). Eight children (4.9%) were incorrectly classified as not requiring intervention because of a low DSRS score, but the psychosocial counselor rated them with high psychosocial disability scores (false negatives). For the CPSS cutoff score of 20 or above indicating need for intervention, 19 children (11.7%) were true positives, 98 (60.5%) were true negatives, 36 (22.2%) were false positives, and nine (5.6%) were false negatives.

Tables 4 and 5 list the psychometric properties based on individual items of the DSRS and CPSS respectively. The two items related to gastrointestinal issues (stomachaches, DSRS.6; enjoying food, DSRS.8) had the lowest inter-item correlation suggesting that they were least associated with other items on the instrument. Feeling lonely (DSRS.15) had the strongest inter-item correlation. Only three items individually distinguished the indication-to-treat versus no-indication-to-treat groups after Bonferonni-type corrections: having lots of energy (DSRS.7, which is recoded inversely when calculating total score), life is not worth living (DSRS.10), and feeling lonely (DSRS.15).

**Table 4:** Depression Self Rating Scale: individual item psychometrics

	No Indication to Treat (n = 134)		Indication to Treat (n = 28)				
					
Item	Mean	SD	Mean	SD	Inter-item Correlation	T-test	Adj. *p*-value*
1. Look forward to things	1.46	0.54	1.39	0.57	0.14	0.60	*NS*

2. Sleep	1.69	0.48	1.39	0.57	0.30	2.56	0.27

3. Crying	0.87	0.45	1.18	0.48	0.30	3.12	0.05

4. Like playing	1.37	0.61	1.32	0.55	0.16	0.45	*NS*

5. Running away	0.13	0.38	0.29	0.53	0.14	1.42	*NS*

6. Tummy aches	0.99	0.42	1.11	0.57	0.06	1.08	*NS*

7. Lots of energy	1.43	0.50	1.07	0.38	0.33	4.25	0.00

8. Enjoy food	1.26	0.49	1.18	0.55	0.09	0.74	*NS*

9. Stick up for self	1.25	0.54	1.07	0.60	0.42	1.42	*NS*

10. Not worth living	0.42	0.57	0.96	0.74	0.40	3.67	0.02

11. Good at things	1.37	0.52	1.36	0.56	0.22	0.14	*NS*

12. Enjoy things	1.46	0.50	1.29	0.46	0.16	1.82	*NS*

13. Talking with family	1.82	0.38	1.46	0.64	0.29	2.85	0.14

14. Bad dreams	1.01	0.42	1.32	0.48	0.23	3.16	0.05

15. Feel lonely	0.35	0.54	0.82	0.67	0.52	3.49	0.02

16. Easily cheered up	1.18	0.75	0.79	0.88	0.22	2.21	0.59

17. Unbearable sadness	0.88	0.56	1.21	0.63	0.34	2.60	0.25

18. Bored (disinterested)	0.61	0.61	1.00	0.61	0.31	3.07	0.07

* *p*-value corrected for 18 tests using Bonferroni-type corrections. Adjusted p-values are only presented for those items with significant unadjusted p-values. *NS *refers to nonsignificant unadjusted *p*-values.

**Table 5:** Child PTSD Symptom Scale: individual item psychometrics

	No Indication to Treat (n = 134)		Indication to Treat (n = 28)				
					
Item	Mean	SD	Mean	SD	Inter-item Correlation	T-test	Adj. *p*-value*
1. Intrusive thoughts	1.32	0.62	1.54	0.69	0.47	1.52	*NS*

2. Nightmares	1.07	0.60	1.43	0.50	0.34	3.27	0.03

3. Flashbacks	0.93	0.62	1.46	0.69	0.54	3.76	0.02

4. Distress with reminders	1.13	0.64	1.39	0.74	0.60	1.77	*NS*

5. Somatic distress	1.04	0.69	1.36	0.62	0.53	2.43	0.32

6. Avoid feelings	1.36	0.70	1.61	0.57	0.54	2.03	0.83

7. Avoid activities	1.40	0.76	1.54	0.69	0.55	0.91	*NS*

8. Amnesia	1.03	0.72	1.61	0.50	0.63	5.11	0.00

9. Less interest in activities	0.97	0.56	1.07	0.66	0.46	0.75	*NS*

10. Not close to people	0.37	0.58	0.75	0.93	0.33	2.11	0.73

11. No strong feelings	0.55	0.60	1.00	0.72	0.49	3.08	0.07

12. Foreshortened future	0.78	0.69	1.46	0.79	0.40	4.22	0.00

13. Sleep difficulties	0.91	0.72	1.29	0.76	0.49	2.39	0.37

14. Irritable/angry	0.81	0.65	1.29	0.71	0.45	3.28	0.03

15. Concentration problems	0.84	0.54	1.18	0.72	0.45	2.38	0.39

16. Overly careful	0.82	0.60	1.11	0.74	0.46	1.93	*NS*

17. Easily startled	1.14	0.62	1.50	0.58	0.37	2.95	*NS*

* *p*-value corrected for 17 tests using Bonferroni-type corrections. Adjusted p-values are only presented for those items with significant unadjusted p-values. *NS *refers to nonsignificant unadjusted *p*-values.

On the CPSS, the two items strongly correlated with others were distress with reminders (CPSS.4) and traumatic amnesia (CPSS.8). After making Bonferroni-type corrections, five items individually distinguished between indication for treatment and no indication for treatment groups: nightmares (CPSS.2), flashbacks (CPSS.3), amnesia (CPSS.8), feelings of foreshortened future (CPSS.12), and angry at small matters (CPSS.14).

## Discussion

We proposed six questions in the introduction that can be used by mental health researchers and psychosocial interventionists working with children in cross-cultural settings. Our goal was to use these six questions to evaluate our process of adapting and validating instruments for mental health and psychosocial research with children affected by armed conflict in Nepal. For the first question "*What is the purpose of the instrument?*", our goals were evaluating treatment, estimating prevalence, and detecting MHPS-related disability. For the second question "*What is the construct to be measured?*", the purpose dictated employing cross-cultural constructs that were locally salient and sufficiently resembling the psychiatric categories of depression and PTSD. Therefore, we required a validation against an external criterion related to diagnosis and impairment, which we accomplished through ratings on structured interviews with the GAPD and K-SADS completed by a psychosocial counselor. However, before the validation we needed to assure appropriate transcultural translation by answering questions three, four, and five.

For the third question, "*What are the contents of the construct?*", somatic symptoms stood out as lacking content equivalence between Western populations and Nepali populations. Qualitatively, children did not associate DSRS items six and eight (appetite loss and stomachaches) with sadness or depression. Quantitatively, these items had no significant discriminant validity and the lowest inter-item correlations. Similarly, during validation of the Beck Depression Inventory and Beck Anxiety Inventory for adult populations in Nepal, gastrointestinal complaints did not differentiate between persons with and without psychological distress [58-60]. For future research, we would recommend exploring the discriminant validity of other somatic complaints such as headaches or paresthesia, in place of gastrointestinal complaints. In place of an appetite question, more concrete items about changes in food eaten regardless of food availability may be more effective, e.g. "Have you been eating less food than usual over the past week even when food was available?" or "Have family members said that you are not eating enough food?" Alternatively, these items could be dropped from the DSRS. Based on current evidence, questions of abdominal complaints and appetite changes do not help identify depression among children or adults in Nepal.

In the CPSS, children in focus groups identified two items as common responses to trauma that were not associated with distress. These items were avoiding activities and people related to the event (CPSS.7) and less interest in activities (CPSS.9). In a conflict zone, it would be appropriate to avoid a place where an attack or bombing occurred or avoid people in uniform who may incite violence. Similarly, children added the common cultural explanation that ghosts and spirits haunt places where bad events happened. Quantitatively, these items also had poor discriminant validity. In settings of recent conflict, items related to avoidance and changed activities may not reflect pathology and could erroneously inflate PTSD prevalence estimates. However, these items may be more salient in detecting MHPS problems with greater time after cessation of political violence.

A surprising finding was the disjoint between the focus group qualitative findings and the validation study in regards to traumatic amnesia (CPSS.8). Children in multiple focus groups stated that traumatic amnesia does not occur. Moreover, they stated that their goal was *to forget *the event, and forgetting leads to feeling better. In the Nepali context, forgetting does not literally refer to being unable to remember but rather refers to not having intrusive memories [36]. The denial of traumatic amnesia is consistent with qualitative work among adult trauma survivors in Nepal who claimed that they remember all the details of their traumatic events but wish they could not [35]. However, when the 162 children completed the CPSS, traumatic amnesia had greater mean endorsement than ten of the other items. Traumatic amnesia also had the greatest inter-item correlation and had significant individual item discriminant validity. This raises questions about why these qualitative and quantitative responses appear to be at odds. To resolve this, it would be helpful to do more qualitative work to find out why some children endorsed the traumatic amnesia item. The issue of traumatic brain injury may be salient here because many child soldiers with PTSD reported exposure to bomb blasts, which could affect trauma recall [27].

For the fourth question "*What are the idioms used to identify specific items?*", the wording was changed on numerous items. To improve acceptability of the instruments, children suggested the removal of language that appeared to blame and stigmatize respondents. In Nepal, it is common to view traumatic experiences as the result of bad karma [35]. Therefore, children suggested removing the mention of guilt in the CPSS because it could be perceived as reinforcing blame among trauma survivors.

For the fifth question, "*How should questions and responses be structured?*", it was culturally unfamiliar to present children with declarative statements to endorse the degree of veracity. It was more understandable to administer the items as questions. In addition, the order of the response set on the DSRS was counter-intuitive. A striking finding was children's interpretation of locally developed pictographic representation of emotional gradations. The *dhoko*-basket scale had a different meaning to the children than that intended by the researchers. An intended physical to emotional abstraction was interpreted instead as an economic to emotional abstraction. This illustrates how attempts at cultural-adaptation can lead to even greater confusion or misrepresentation. It is as important to do focus groups about locally-developed items and responses sets as it is to assess Western-developed tools.

Regarding question six, "*What does the instrument score mean?*", we found that the DSRS correctly classified 79% of children: 12% of children were correctly classified as having high DSRS scores and having counselor rated psychosocial disability, and 67% were correctly classified as having low DSRS scores and lacking counselor rated psychosocial disability. Of the remaining 21% who were incorrectly classified, the majority (16%) had high DSRS scores but lacked counselor rated psychosocial disability (false positives). This is reflected in the moderate sensitivity, specificity, and negative predictive value contrasted with the poor positive predictive value (PPV). The low PPV is influenced by the low prevalence of psychosocial disability in this specific sample; only 28 of 134 children were rated with high GAPD scores. If the overall population also has a low prevalence, then the DSRS, if used as a screening tool, would lead to enrollment of approximately two children without psychosocial disability for every one with psychosocial disability (one true positive for every two false positives). That said, the instrument performs well at minimizing the number of children who would be left out of an intervention (few false negatives). With the DSRS, less than five percent of children would be mistakenly excluded from a support program.

The CPSS performs similarly: 72.2% of children are correctly classified. However, nearly one quarter are misclassified with high CPSS scores but lack counselor rated psychosocial disability (false positive). Of the total sample, only 5.6% of children have psychosocial disability but are misclassified with low CPSS scores (false negatives). Low prevalence of trauma-related disability also contributed to the large difference between the negative and positive predictive values. If psychosocial disability were more prevalent in the sample, the instrument would have shown greater positive predictive value. Ultimately, both the DSRS and CPSS perform well to include the majority of children in need of services. However, the instruments, if used as screening tools, would include a large number of children who do not have psychosocial disability, thus reducing the cost effectiveness of a resource-intensive intervention.

Using an adapted validation procedure with a psychosocial counselor who received extra training was a useful alternative to clinician-rated validation procedures in a setting without child mental health specialists. Our procedure did not require that the few psychiatrists or psychologists in Nepal leave their obligations of providing care. It did not incur the high cost of purchasing expert clinician's time. It also is replicable for other validation procedures. In settings similar to Nepal, highly trained psychosocial workers with multiple years of experience may be best positioned to make assessments on indication for psychosocial treatment because they are the individuals with the greatest training and experience in this setting, and they know the cultural context. Moreover, the emphasis on psychosocial disability using a structured modification of the GAPD assured that the validated instruments captured children with functioning problems and not only presence of symptoms. Validation of the Child Psychosocial Distress Screener in Burundi employed a similar approach [16]. Ultimately, our alternative procedure produced instruments with acceptable psychometric properties. When compared with Birleson's [45] original validation of the DSRS, the Nepali DSRS has similar sensitivity (English 69% vs. Nepali 71%) but better specificity (English 57% vs. Nepali 81%).

We were surprised to find that item discriminant validity varied significantly between the Nepali CPSS and the English CPSS psychometrics established in the U.S. [50]. In the original U.S. sample, the six items with lowest discriminant validity included traumatic amnesia and foreshortened future--items that showed the strongest validity in this Nepali sample. Moreover, the three items that showed the strongest validity in the American youth sample performed poorly in the Nepali sample: distress with reminders, less interest in activities, and overly careful. It is unclear whether this is due to the nature of trauma studied--a single earthquake in California versus a decade of war in Nepal--or other cultural differences. In another U.S. sample, the irritability/anger item was associated strongly with disability, which is in keeping with the Nepali findings [61].

The study also highlights items that could be selected to produce brief screening versions of the DSRS and CPSS. From the DSRS, the three items with significant discriminant validity for indication-to-treat were having energy to complete daily activities (DSRS.7), feeling that life's not worth living (DSRS.10), and feeling lonely (DSRS.15). The "life not worth living" item is important to include also because affirmative responses should trigger a suicide screening and referral for services. Five items on the CPSS had significant discriminant validity: nightmares (CPSS.2), flashbacks (CPSS.3), traumatic amnesia (CPSS.8), feelings of a foreshortened future (CPSS.12), and easily irritated/angered at small matters (CPSS.14). Interestingly, these five items comprise two items from criterion B (re-experiencing), two items from criterion C (avoidance/numbing), and one from criterion D (increased arousal) of the DSM-IV PTSD diagnostic criteria.

Future studies and transcultural translation/validation studies in other setting could improve upon the work described here. After development of an instrument, it would be helpful to do cognitive interviewing with a subset of children. Cognitive interviewing is a qualitative research method in which questionnaire respondents are asked how they interpret a question and why they provide a specific response [62]. This is a more individualistic approach to complement similar information obtained through focus groups. This would help to elucidate contradictory findings such as that related to traumatic amnesia. Furthermore, a larger sample size for the validation study would have increased the power for individual item discrimination. All of the psychosocial functioning assessments were done by one psychosocial counselor. With more assessors, inter-rater reliability could have been assessed and idiosyncrasies of individual raters may be revealed.

Before concluding, it is helpful to consider how validated instruments can be used to shed light on cost-effectiveness of an intervention. Psychosocial practitioners and researchers increasingly have argued against providing interventions purely based on membership in a vulnerable group, such as being a former child soldier or a victim of child trafficking [10,63,64]. Rather, evaluation of a child's MHPS is needed to determine which children may need intervention including children who are not members of a target group. Providing interventions to only children in a specific group can worsen stigma and decrease community support for a program and its beneficiaries.

Screening with validated instruments is an alternative to group-based selection for an intervention [65]. Based on our findings with the DSRS and CPSS, we can compare how the instruments would perform in a cost-based analysis using a screening based strategy. If a specific intervention in LAMIC cost $20 per child [65] and the DSRS were used to screen children for the intervention, the actual cost per child would be higher because of the number of false positives. With the DSRS, there were 26 false positive and 20 true positives who scored above the DSRS cutoff. Therefore, 1.3 healthy children would be treated for every child with depression identified, resulting in 2.3 children (1.3 healthy children + 1 depressed child) entering an intervention. The intervention that cost $20 would then cost $46 (2.3 × $20) to treat one depressed child. For the CPSS, there were 19 true positives and 36 false positives; 1.9 healthy children would be recruited into an intervention for every one psychologically traumatized child at the CPSS cutoff of 20. Therefore, a psychosocial intervention costing $20 per child would cost $58 (2.9 × $20) in programmatic expenses because of the need to include 1.9 healthy children in addition to every traumatized child. Additional calculations are required to estimate the costs to society of not including children with MHPS problems in an intervention. For example, if a child does not receive the intervention, what are the reductions in productive labor, increases in crime, and increases in other healthcare costs. Both the DSRS and CPSS have good psychometric properties (high negative predictive value) to minimize the number of children mistakenly excluded from an intervention, and therefore minimize the costs to society of not treating an individual. The more expensive the intervention, the more crucial it is to have instruments that have strong psychometric properties to properly include and exclude children in psychosocial programs. With the majority of child MHPS programs in LAMIC not employing validated instruments, there is substantial risk of economic inefficiency and financial waste through the inappropriate inclusion or exclusion of beneficiaries.

## Conclusions

Validation of instruments in LAMIC, where the majority of the world's children live, is crucial for the advancement of research and intervention for children. Our findings highlight the potential pitfalls of assuming that only translation and back-translation can capture cultural differences in performance of mental health instruments. While the specific process of transcultural translation and validation will vary based on objectives and local resources, a critical evaluation of the translation and validation process is indispensible. The six questions outlined here provide a framework for researchers and interventionists to systematically do such an evaluation of tools. In addition, the study demonstrated that task-shifting the validation process to trained non-psychiatrists using a structured interview can produce acceptable psychometric properties. Validated instruments additionally are crucial to maximize the financial benefit for improving child psychosocial wellbeing and minimize the misapplication of economic resources in LAMIC. Only through the appropriate development and interpretation of instrument-derived findings do we reduce the risk of producing misleading conclusions and generalizations about the mental health of the majority of the world's children.

## Competing interests

The authors declare that they have no competing interests.

## Authors' contributions

BK and MJ designed the study, trained and supervised the psychosocial counselor conducting structured interviews, supervised the qualitative research and analyses, conducted statistical analyses, and drafted the manuscript. WT participated in designing the study and drafting the manuscript. NL conducted focus groups, supervised the training, administration, and data entry of survey responses, participated in the statistical analyses, and reviewed final translations. SM conducted focus groups, coded qualitative findings, and reviewed final translations. NU participated in translation, conducting focus groups, and review of qualitative findings. All authors read and approved the final manuscript.

## Pre-publication history

The pre-publication history for this paper can be accessed here:

http://www.biomedcentral.com/1471-244X/11/127/prepub

## Supplementary Material

Additional file 1 **Nepali versions of the DSRS and CPSS**. The final Nepali language translations of the Depression Self Rating Scale (DSRS) and Child PTSD Symptom Scale (CPSS). Click here for file

Additional file 2 **English back-translations of Nepali DSRS and CPSS**. The final English back-translations of the Depression Self Rating Scale (DSRS) and Child PTSD Symptom Scale (CPSS) are compared with the original English items on the DSRS and CPSS. Comments are provided explaining changes in wording, use of probes, and structuring of items. Click here for file
