# Supplementary material for: Validation of cross-cultural child mental health and psychosocial research instruments: adapting the Depression Self-Rating Scale and Child PTSD Symptom Scale in Nepal
Source: BMC Psychiatry. 2011 Aug 4;11:127. doi: 10.1186/1471-244X-11-127 (PMC3162495; doi:10.1186/1471-244X-11-127)
Supplement: Additional file 1 — Nepali versions of the DSRS and CPSS. The final Nepali language translations of the Depression Self Rating Scale (DSRS) and Child PTSD Symptom Scale (CPSS). [file 1471-244X-11-127-S1.PDF]

APPENDICES

Appendix 1. Depression Self Rating Scale (DSRS), (Birleson, 1981) – Nepali Version

DSRS 1: तपाई पहिले जति चिजहरु वा कुराहरु चाहनु हुन्थ्यो अहिले पनि त्यति नै चाहनु हुन्छ ?(उदाहरणको लागि तपाई पहिले मामाघर जानु हुँदा जति खुशी हुने गर्नु हुन्थ्यो अहिले पनि मामाघर जाने भन्दा त्यति नै खुसी हुने गर्नु हुन्छ)
पहिले जति पटक्कै चाहन्न........................०
पहिलेको भन्दा कम चाहन्छु.......................१
पहिलेको जत्तिकै चाहन्छु..........................२

DSRS 2: तपाई कत्तिको राम्रोसँग सुत्न (निदाउन) सक्नु हुन्छ ?
कहिल्यै पनि निदाउन सक्दिन..................०
कहिलेकाही निदाउन सक्छु........................१
सधैजसो निदाउन सक्छु..........................२

DSRS 3: तपाईलाई कत्तिको रुन मन लाग्छ ?
कहिल्यै पनि रुन मन लाग्दैन.................०
कहिलेकाही रुन मन लाग्छ......................१
धेरैजसो रुन मन लाग्छ..........................२

DSRS 4: तपाईलाई कत्तिको खेल्न मन लाग्छ ?
कहिल्यै पनि खेल्न मन लाग्दैन................०
कहिलेकाहि खेल्न मन लाग्छ....................१
धेरैजसो खेल्न मन लाग्छ.......................२

DSRS 5: तपाईलाई कत्तिको भाग्न मन लाग्छ ? (जहाँबाट पनि जस्तैः स्कुल, घर, खेल्ने ठाँउ आदि)
कहिल्यै पनि भाग्न मन लाग्दैन.............०
कहिलेकाहि भाग्न मन लाग्छ.................१
सधैजसो भाग्न मन लाग्छ.....................२

DSRS 6: तपाईको पेट कत्तिको दुख्ने गर्छ ?
कहिल्यै पनि दुख्दैन...........................०
कहिलेकाहि दुख्छ...............................१
सधैजसो दुख्छ.................................२

DSRS 7: तपाईलाई आफु कत्तिको जाँगरिलो छु जस्तो लाग्छ ? (उदाहरणको लागि धेरैबेर काम गर्दा पनि नथाक्ने वा धेरैबेर पढ्न सक्ने आदि)
कहिल्यै पनि लाग्दैन.......................०
कहिलेकाहि लाग्छ............................१
सधैजसो लाग्छ.............................२

DSRS 8: तपाईलाई खानेकुरा देख्दा कत्तिको खाँउखाँउ लाग्छ ?
कहिल्यै पनि खाँउखाँउ लाग्दैन.............०
कहिलेकाहि खाँउखाँउ लाग्छ.................१
सधैजसो खाँउखाँउ लाग्छ...................२

DSRS 9: कसैले तपाईलाई वा अरु कसैलाई हेप्दा वा होच्याउदा उहाँलाई तपाईले आफ्नो कुरा कत्तिको बुझाउन सक्नु हुन्छ ?
कहिल्यै पनि सक्दिन........................०
कहिलेकाहि सक्छु............................१
सधैजसो सक्छु..............................२

DSRS 10: तपाईलाई आफ्नो जीवन कत्तिको बेकार जस्तो लाग्छ ?
कहिल्यै पनि बेकार लाग्दैन..............०
कहिलेकाहि बेकार लाग्छ..................१
सधैजसो बेकार लाग्छ.....................२

DSRS 11: तपाईलाई आफुले गरेको कामहरु कत्तिको राम्रो लाग्छ ?
कहिल्यै पनि राम्रो लाग्दैन..............०
कहिलेकाहि राम्रो लाग्छ.................१
सधैजसो राम्रो लाग्छ....................२

DSRS 12: आफुले गरेको कामकुराहरुमा तपाई पहिले जति खुसी हुनुहुन्थ्यो अहिले पनि त्यति नै खुसी हुने गर्नु हुन्छ ?
कहिल्यै पनि खुसी हुन्न..................०
कहिलेकाहि खुसी हुन्छु.....................१
सधैजसो खुसी हुन्छु.......................२

DSRS 13: तपाईलाई आफनो परिवारसँग कुराकानी गर्न कत्तिको मन लाग्छ ?
कहिल्यै पनि मन लाग्दैन..............०
कहिलेकाहि मन लाग्छ...................१
धेरैजसो मन लाग्छ.....................२

DSRS 14: तपाईले नराम्रा वा डरलाग्दा सपनाहरु कत्तिको देख्ने गर्नु हुन्छ ?
कहिल्यै पनि देख्दिन......................०
कहिलेकाहि देख्छु..........................१
सधैजसो देख्छु.............................२

DSRS 15: तपाईलाई आफु कत्तिको एक्लो छु जस्तो लाग्छ?
कहिल्यै पनि एक्लो लाग्दैन............०
कहिलेकाहि एक्लो लाग्छ................१
सधैजसो एक्लो लाग्छ..................२

DSRS 16: तपाईलाई दुःख लागेपछि खुसी हुनलाई कत्तिको समय लाग्छ ?
धेरै समय लाग्छ..........................०
अलिअलि समय लाग्छ..................१
छोटो समय लाग्छ........................२

DSRS 17: तपाईलाई सहनै नसक्ने गरी (असाध्यै) कत्तिको दुःख लाग्छ ?
कहिल्यै पनि दुःख लाग्दैन.............०
कहिलेकाहि दुःख लाग्छ.................१
सधैजसो दुःख लाग्छ....................२

DSRS 18: तपाईलाई धेरै कुरामा आफ्नो ईच्छा हराएको जस्तो लाग्छ ? (जस्तै : रुचि, रहर वा चाहना)
कहिल्यै पनि लाग्दैन.....................०
कहिलेकाहि लाग्छ.........................१
सधैजसो लाग्छ............................२

Appendix 2. Child PTSD Symptom Scale (CPSS), (Foa et al 2001) – Nepali Version

अब म बालवालिकाहरुले दु:खद घटना पछि के कस्ता समस्याहरु अनुभव गर्दछन भन्ने बारेमा केहि प्रश्नहरु सोध्न गईरहेको छु।

CPSS-A तपाईको जीवनमा आईपरेको सबभन्दा दु:खद घटना कुन होला ?
(उदाहरणको लागि तपाईको परिवारका सदस्यहरुलाई कसैले हातपात गरेको आफ्नै आँखाले देखेको, कसैलाई आक्रमण गरेको देखेको, क्रुर यातना दिएको देखेको , अपहरण गरेको देखेको, बम खसालेको देखेको, गोली हानाहान भएको देखेको वा घरहरु जलिरहेको देखेको आदि घटनाहरु घटेको छ कि छैन भनेर विद्यार्थीलाई सोध्न सकिन्छ ।)

____

____

____

CPSS-B त्यो घटना कहिले भएको थियो ?__________________

अब म तपाईले भर्खरै भनेको दु:खद घटना वा अन्य कुनै घटनाहरु गएको दुई हप्तामा तपाईको मनमा कत्तिको संझना आए वा त्यस्ता घटनाले तपाईलाई कत्तिको दु:ख दिए होला भन्ने बारेमा केहि प्रश्नहरु सोध्न गईरहेको छु। म तपाईलाई प्रश्नहरु र त्यसका सम्भावित उत्तरहरु पढेर सुनाउने छु र तपाईले आफुलाई मिल्ने उत्तर छान्नु पर्ने छ।

CPSS 1 तपाईले नचाहँदा नचाहदै पनि पहिले घटेका घटनाहरुको दु:ख दिने तस्वीर, सोच वा यादहरु (सम्झना) कत्तिको आए होला ?
कहिल्यै पनि आएन...०
कहिलेकाहि आए (हप्तामा एक पटक)..............................१
धेरैजसो आए (हप्तामा २ देखि ४ पटक)........................२
सधैजसो आए (हप्तामा ५ पटक वा सो भन्दा धेरै)..........३

CPSS 2 तपाईले कति पटक नराम्रा वा डरलाग्दा सपनाहरु देख्नु भयो होला ?
कहिल्यै पनि देखिन...०
कहिलेकाहि देखे (हप्तामा एक पटक)................................१
धेरैजसो देखे (हप्तामा २ देखि ४ पटक)...........................२
सधैजसो देखे (हप्तामा ५ पटक वा सो भन्दा धेरै)..............३

CPSS 3 तपाईलाई आफु फेरी त्यही घटनामा परेजस्तो, केही सुने जस्तो र केही देखे जस्तो कत्तिको भएको थियो ?
कहिल्यै पनि भएन...०
कहिलेकाहि भयो (हप्तामा एक पटक)..................................१
धेरैजसो भयो (हप्तामा २ देखि ४ पटक)...........................२
सधैजसो भयो (हप्तामा ५ पटक वा सो भन्दा धेरै)..............३

CPSS 4 तपाईलाई पहिले घटेका घटनाको बारेमा सोच्दा वा सुन्दा, दु:ख लाग्ने, तर्सिने र रिस उठ्ने कत्तिको भयो होला ?
कहिल्यै पनि भएन...०
कहिलेकाहि भयो (हप्तामा एक पटक)...................................१
धेरैजसो भयो (हप्तामा २ देखि ४ पटक)..............................२
सधैजसो भयो (हप्तामा ५ पटक वा सो भन्दा धेरै)................३

CPSS 5 तपाईलाई पहिलेका घटनाको बारेमा सोच्दा वा सुन्दा तपाईको शरीरमा कत्तिको असर पर्‍यो ? (
उदाहरणको लागि मुटु ढुकढुक हुने, सास फेर्न गाह्रो हुने, शरीरमा खलखल पसिना आउने वा काप्ने)
कहिल्यै पनि परेन..०
कहिलेकाहि पर्‍यो (हप्तामा एक पटक)...................................१
धेरैजसो पर्‍यो (हप्तामा २ देखि ४ पटक)...............................२
सधैजसो पर्‍यो (हप्तामा ५ पटक वा सो भन्दा धेरै)..................३

CPSS 6 तपाईले पहिले घटेका घटनाहरुबारे नसोच्ने वा कुरा नगर्न कत्तिको कोशिस गर्नु भयो ?
कहिल्यै पनि कोशिस गरिन...०
कहिलेकाहि कोशिस गरे (हप्तामा एक पटक)..............................१
धेरैजसो कोशिस गरे (हप्तामा २ देखि ४ पटक)......................२
धेरैजसो कोशिस गरे (हप्तामा ५ पटक वा सो भन्दा धेरै)........३

CPSS 7 तपाई पहिले घटना घटेका ठाँउमा नजाने अथवा घटनासँग सम्बन्धित मान्छेलाई नभेट्ने वा घटना सम्बन्धि क्रियाकलाप नगर्ने कत्तिको कोशिस गर्नु भयो होला ?
कहिल्यै पनि कोशिस गरिन...०
कहिलेकाहि कोशिस गरे (हप्तामा एक पटक)..........................१
धेरैजसो कोशिस गरे (हप्तामा २ देखि ४ पटक).......................२
सधैजसो कोशिस गरे (हप्तामा ५ पटक वा सो भन्दा धेरै ३

CPSS 8 तपाईलाई पहिलेका दु:खदायी घटनाको महत्वपुर्ण कुराहरु सम्झन कत्तिको गाह्रो भएको थियो ?
(उदाहरणको लागि तपाईको भाईलाई केही महिना अगाडि कसैले कुटेको तपाईले देख्नु भएको थियो । अहिले तपाईलाई उक्त मान्छेको अनुहार सम्झन कत्तिको गाह्रो भएको थियो ।)
कहिल्यै पनि गाह्रो भएन...०
कहिलेकाहि गाह्रो भयो (हप्तामा एक पटक)..........................१
धेरैजसो गाह्रो भयो (हप्तामा २ देखि ४ पटक)..................२
सधैजसो गाह्रो भयो (हप्तामा ५ पटक वा सो भन्दा धेरै)......३

CPSS 9 तपाईलाई सधैं गर्न मन लागेका कामहरु कत्तिको गर्न मन लागेन होला ?
कहिल्यै पनि मन लागेन..०
कहिलेकाहि मन लागेन (हप्तामा एक पटक) १
धेरैजसो मन लागेन (हप्तामा २ देखि ४ पटक) २
सधैजसो मन लागेन (हप्तामा ५ पटक वा सो भन्दा धेरै)....३

CPSS 10 तपाईको मनमा साथीभाईबाट वा परिवारबाट कत्तिको टाढा भएको जस्तो लाग्छ होला ?
कहिल्यै पनि लागेन..०
कहिलेकाहि लाग्यो (हप्तामा एक पटक) १
धेरैजसो लाग्यो (हप्तामा २ देखि ४ पटक).....................२
सधैजसो लाग्यो (हप्तामा ५ पटक वा सो भन्दा धेरै)........३

CPSS 11 तपाईलाई सुख भएको बेला पनि मन खुसी नहुने, दु:ख भएको बेला पनि मन दु:खी नहुने कत्तिको भएको थियो ? (
उदाहरणको लागि : मानौ तपाई अहिले रमाईलो भईरहेको ठाँउमा हुनु हुन्छ तपाईका सबै साथीहरु रमाईलो गरिरहेका छन् तर तपाईलाई भने केहिपनि रमाईलो लागेको छैन)
कहिल्यै पनि भएन...०
कहिलेकाहि भयो (हप्तामा एक पटक)...........................१
धेरैजसो भयो (हप्तामा २ देखि ४ पटक)......................२
सधैजसो भयो (हप्तामा ५ पटक वा सो भन्दा धेरै).........३

CPSS 12 तपाईलाई आफुले चाहेका कुरा भविष्यमा पुरा हुदैनन् कि जस्तो कत्तिको लागेको थियो ? *(जस्तैः जागिर नपाउने, पढाई पुरा गर्न नसक्ने)*
कहिल्यै पनि लागेन..०
कहिलेकाहि लाग्यो (हप्तामा एक पटक)........................१
धेरैजसो लाग्यो (हप्तामा २ देखि ४ पटक)...................२
सधैजसो लाग्यो (हप्तामा ५ पटक वा सो भन्दा धेरै)......३

CPSS 13 तपाईलाई राम्ररी निन्द्रा नलाग्ने र राति ब्युझने कति पटक भयो होला ?
कहिल्यै पनि भएन..०
कहिलेकाहि भयो (हप्तामा एक पटक).........................१
धेरैजसो भयो (हप्तामा २ देखि ४ पटक).....................२
सधैजसो भयो (हप्तामा ५ पटक वा सो भन्दा धेरै)........३

CPSS 14 तपाईलाई सानो कुरामा पनि रिस उठ्ने अनि झर्को लाग्ने कति पटक भयो होला ?
कहिल्यै पनि भएन...०
कहिलेकाहि भयो (हप्तामा एक पटक).......................१
धेरैजसो भयो (हप्तामा २ देखि ४ पटक)................२
सधैजसो भयो (हप्तामा ५ पटक वा सो भन्दा धेरै).....३

CPSS 15 तपाईलाई आफुले गरिरहेको काममा ध्यान दिन नसक्ने कत्तिको भएको थियो ? *(उदाहरणको लागि पढेको कुरा छिटो बिर्सने, सरले पढाईराखेको बेला ध्यान दिन नसक्ने आदि)*
कहिल्यै पनि भएन...०
कहिलेकाहि भयो (हप्तामा एक पटक).......................१
धेरैजसो भयो (हप्तामा २ देखि ४ पटक).................२
सधैजसो भयो (हप्तामा ५ पटक वा सो भन्दा धेरै).....३

CPSS 16 तपाईलाई कत्तिको आफुलाई बढी शंका लागे जस्तो अथवा चाहिने भन्दा बढी होशियार भएको जस्तो भएको थियो ? *(जस्तै धेरै पटक ढोका लगाएको छ की छैन भनेर हेर्नु, भोज भतेरमा जाँदा कसैले आफुलाई हेरिरहेको जस्तो लाग्नु, कोहि पछि लागे जस्तो लाग्नु आदि)*
कहिल्यै पनि भएन...०
कहिलेकाहि भयो (हप्तामा एक पटक).....................१
धेरैजसो भयो (हप्तामा २ देखि ४ पटक)................२
सधैजसो भयो (हप्तामा ५ पटक वा सो भन्दा धेरै)....३

CPSS 17 तपाईलाई कति पटक सानो कुरामा पनि झस्किने, एक्कासी कुनै आवाज सुन्दा झसंग हुने कत्तिको भएको थियो ?
कहिल्यै पनि भएन..०
कहिलेकाहि भयो (हप्तामा एक पटक)......................१
धेरैजसो भयो (हप्तामा २ देखि ४ पटक)................२
सधैजसो भयो (हप्तामा ५ पटक वा सो भन्दा धेरै).....३
