# Supplementary material for: Validation of cross-cultural child mental health and psychosocial research instruments: adapting the Depression Self-Rating Scale and Child PTSD Symptom Scale in Nepal
Source: BMC Psychiatry. 2011 Aug 4;11:127. doi: 10.1186/1471-244X-11-127 (PMC3162495; doi:10.1186/1471-244X-11-127)
Supplement: Additional file 2 — English back-translations of Nepali DSRS and CPSS. The final English back-translations of the Depression Self Rating Scale (DSRS) and Child PTSD Symptom Scale (CPSS) are compared with the original English items on the DSRS and CPSS. Comments are provided explaining changes in wording, use of probes, and structuring of items. [file 1471-244X-11-127-S2.PDF]

ADDITIONAL FILE 2 – Back-translations of DSRS and CPSS

Table 1. Depression Self Rating Scale (DSRS): Original items, back-translation of final Nepali items, and comments from children's focus groups.

1.	*Original Item*	I look forward to things as much as I used to
	Back-translation	"Do you want at present all the things that you have wanted in the past? (For example, you enjoyed going to your maternal uncle's house before, but now you no longer enjoy it.)"
	Comments	The example of going to visit one's maternal uncle "*maamaa ghaar*" was added because this is a common enjoyable activity for children.
2.	*Original Item*	I sleep very well
	Back-translation	"Are you able to sleep well?"
	Comments	All children understood this item and said that when one is sad *"dukkha laagyo*" they may have problems sleeping. Children understood "sleep well" as "sleeping nicely and comfortably, and having good dreams."
3.	*Original Item*	I feel like crying
	Back-translation	"How much do you feel like weeping?"
	Comments	Children stated, "when I have been hurt badly, I feel like crying very much." Another child stated, "when [our family member] died, I felt like crying endlessly."
4.	*Original Item*	I like to go out to play
	Back-translation	"How much do you feel like playing?"
	Comments	Children explained that when they are playing they are happy and do not have other worries.
5.	*Original Item*	I feel like running away
	Back-translation	"How much do you like fleeing away? (Such as run from school, home, or playground.)"
	Comments	Children explained that when they have problems, they feel like running away. They added that "running away is a bad way to solve problems."
6.	*Original Item*	I get tummy aches
	Back-translation	"How much do you feel stomachaches?"
	Comments	Children understood this question. However, they felt it was a "foolish question" because everyone's stomach hurts. "Stomachaches are easy, everyone can get them." The children did not associate feeling sad with getting a stomachache.
7.	*Original Item*	I have lots of energy
	Back-translation	"How much strength do you feel you have? (For example, to do housework or school work.)"
	Comments	Children understood the association of "thinking too much" and the body losing energy, "A man who thinks too much becomes thin and faints easily." The children proposed qualifiers of not having the "strength/energy" to complete housework or do one's schoolwork.
8.	*Original Item*	I enjoy my food
	Back-translation	"Do you feel like eating when you see food?"
	Comments	Children found the terminology, "enjoy food" implied "playing with one's food," therefore, they preferred, "feel like eating when you see food."
9.	*Original Item*	I can stick up for myself
	Back-translation	"How much do you feel like telling others what you have in your mind (speaking your mind) when others are harassing/ bothering you or someone else?"
	Comments	The idiom "stick up for yourself" was difficult for children to understand. Therefore, the specific example of telling others what you think when they are doing something inappropriate was added to the question.
10.	*Original Item*	I think life isn't worth living
	Back-translation	"How much do you find that your life is useless?"
	Comments	Children readily understood this question and many endorsed the feeling that their lives were worthless: "It's useless because we don't have any skills, neither do we have good

		friends, nor do we have anybody with us for support. It's not worth trying. What's the use of trying if it's not going to work out?"
11.	*Original Item*<br>*Back-translation*<br>*Comments*	I am good at the things I do<br>“How much do you like the work that you do?”<br>Children replied that "we should feel good about what work we do. We should get satisfaction from that."
12.	*Original Item*<br>*Back-translation*<br>*Comments*	I enjoy things I do as much as I used to<br>“Do you feel as much happiness now as you felt in the work that you did in the past?”<br>Children understood this question.
13.	*Original Item*<br>*Back-translation*<br>*Comments*	I like talking with my family<br>“How much do you feel like talking to your family members?”<br>Children understood this question, and said sharing with family is "a good thing to do."
14.	*Original Item*<br>*Back-translation*<br>*Comments*	I have bad dreams<br>“Do you see bad or frightful dreams?”<br>Children endorsed many bad dreams. Examples included being eaten by a snake, seeing dead people, being chased, falling to one's death, falling from helicopters, falling from trees, feeling death approaching, and being dead.
15.	*Original Item*<br>*Back-translation*<br>*Comments*	I feel very lonely<br>“Do you feel you are alone or lonely?”<br>Children understood this question.
16.	*Original Item*<br>*Back-translation*<br>*Comments*	I am easily cheered up<br>“After you have become sad, does it require a short time for you to be happy?”<br>Children did not understand "easily cheered up," but they stated one should not take a long time to feel happy after feeling sad, for example, "one should only be sad for 2 or 3 minutes, or 5 or 6 minutes, then be happy again."
17.	*Original Item*<br>*Back-translation*<br>*Comments*	I feel so sad I can hardly stand it<br>“Do you feel unbearable sadness?”<br>Children understood "sadness one cannot bear." One child added, "unbearable sadness leads to suicide."
18.	*Original Item*<br>*Back-translation*<br>*Comments*	I feel very bored<br>“Do you feel that you have lost desires or interest in many things?”<br>The term ‘*bore’* is used in Nepali to refer to upset or irritated, therefore "loss of desires and interests" was substituted here.

The Nepali DSRS was adapted from Birleson P: **The Validity of Depressive Disorder in Childhood and the Development of a Self-Rating Scale - a Research Report**. *Journal of Child Psychology and Psychiatry and Allied Disciplines* 1981, **22**(1):73-88.

Table 2. Child PTSD Symptom Scale (CPSS): Original items, back-translation of final Nepali items, and comments from children's focus groups.

1.	*Original Item*	Having upsetting thoughts or images about the event that came into your head when you didn't want them to
	Back-translation	"How much did painful pictures, thoughts, or memories of incidents that occurred in the past come to your brain though you did not want to think of them?"
	Comments	Children discussed that bad memories of people dying do come to mind. They suggested terminology "*yaad*" instead of "*samjhana*" for memory because the latter could refer to other people's memories.
2.	*Original Item*	Having bad dreams or nightmares
	Back-translation	"How many times did you see bad or frightful dreams?"
	Comments	Children stated they have nightmares such as people trying to hurt them with weapons or seeing sick relatives.
3.	*Original Item*	Acting or feeling as if the event was happening again (hearing something or seeing a picture about it and feeling as if I am there again)
	Back-translation	"How many times did you feel that you were involved in the same situation or perhaps heard something or perhaps felt that you saw something?"
	Comments	Children stated they do have feelings of the event happening again, this was referred to as '*jhajhalko aaunchha'*. Children specified that '*jhajhalko aaunchha'* is the sensation of something happening again when in reality it is not happening.
4.	*Original Item*	Feeling upset when you think about it or hear about the event (for example, feeling scared, angry, sad, guilty, etc)
	Back-translation	"How many times did you feel sad or panicked or angry when you thought of the past events?"
	Comments	Children reported that after memories of bad events, they feel fearful or "like a fire in one's heart-mind." The term "guilty" was removed because of implications of blame; it was interpreted as an accusation.
5.	*Original Item*	Having feelings in your body when you think about or hear about the event (for example, breaking out into a sweat, heart beating fast)
	Back-translation	"How much did you experience palpitations, difficulty breathing, heavy perspiration, or shivering heavily when you remembered the past event or heard about it?"
	Comments	Children affirmed "I have done bad things and when I remember them, my heart beats fast, I have difficulty breathing, and I sweat a lot." Children affirmed these somatic terms are understood by both rural and urban children.
6.	*Original Item*	Trying not to think about, talk about, or have feelings about the event
	Back-translation	"How much effort did you make not to think of the past event or not even to talk about it at all?"
	Comments	Children stated that it was good to not think about traumatic events. "If we talk about sad things, we feel miserable. We should forget." The concept of "not having feelings" was not understood as possible by the children, and therefore removed from the item.
7.	*Original Item*	Trying to avoid activities, people, or places that remind you of the traumatic event
	Back-translation	"Did you make an effort not to visit that place of the incident or meet any person related with that incident?"
	Comments	Children stated one should avoid places where bad things happened, and added that this is where ghosts and spirits reside after bad events.
8.	*Original Item*	Not being able to remember an important part of the upsetting event
	Back-translation	"Was it difficult to remember important things of that painful event or situation?"
	Comments	This question was confusing to children because they stated their goal was "to forget" the event. Therefore, "difficulty to remember" was seen as "good" and "nice."
9.	*Original Item*	Having much less interest or doing things you used to do
	Back-translation	"Did you find the work that you found interesting to do in the past was not interesting now?"
	Comments	Children did not say this was a response to bad events. However, the item was left in as it was not considered offensive or inappropriate.
10.	*Original Item*	Not feeling close to people around you

	Back-translation	"How much do you feel that you have been distanced from your friends or family members in your heart-mind?"
	Comments	Children required the phrase "in your heart-mind", otherwise "not feel close" was understood as physical distance, such as moving away from a relative.
11.	*Original Item*	Not being able to have strong feelings (for example, being unable to cry or unable to feel happy)
	Back-translation	"How much did you feel that even when you are comfortable, you are not happy? Or, when you are in sad situations, you did not feel sadness?"
	Comments	Children found the concept of "not able to have strong feelings" difficult to understand (see CPSS#6). Therefore, the example of not feeling happy when friends are happy, or not feeling sad when others are sad was required.
12.	*Original Item*	Feeling as if your future plans or hopes will not come true (for example, you will not have a job or get married or have kids)
	Back-translation	"How much did you feel that what you wished for may not be fulfilled in the future? (For example, you would not be able to get a job or complete your education.)"
	Comments	Children employed a concrete view of traumatic events and implications on one's future? One child gave the example, "If a person is injected by HIV, then he knows he should not marry because the infection is transferrable."
13.	*Original Item*	Having trouble falling or staying asleep
	Back-translation	"How many times did you not have sound sleep or awaken in the middle of the night?"
	Comments	All children understood this question and said insomnia was a common response to bad experiences.
14.	*Original Item*	Feeling irritable or having fits of anger
	Back-translation	"How many times did you feel angry in small matters or get irritated?"
	Comments	Children stated anger was justifiable such as angry at a person who killed a relative or beat the child. Therefore, the qualifier of angry or irritated "in small matters" was added based on suggestions from the children.
15.	*Original Item*	Having trouble concentrating (for example, losing track of a story on the television, forgetting what you read, not paying attention in class)
	Back-translation	"How much did you feel that you were not able to concentrate in the work you were doing? (For example, forgot quickly what you were learning or not concentrate when the teacher was teaching.)"
	Comments	Children endorsed the experience of "not listening properly to what the teacher is saying and forgetting it very quickly" was common after bad experiences. Watching television was removed because it was a luxury item not available to many children.
16.	*Original Item*	Being overly careful (for example, checking to see who is around you and what is around you)
	Back-translation	"How many times did you feel you were more cautious than was necessary, or you were too suspicious? (For example, you checked several times if the door was locked or not, or felt that someone was watching you when you were in public places such as at feasts or parties, or felt that someone was following close behind you?)"
	Comments	Children stated that it was good to be careful, especially in the city or in places of violence. Therefore, children suggested the qualifiers of "more cautious than necessary" or "too suspicious" with examples of checking doors repeatedly or having fear in public places.
17.	*Original Item*	Being jumpy or easily startled (for example, when someone walks up behind you)
	Back-translation	"How many times did you feel startled, or were scared to hear any sound?"
	Comments	Children described that after the murder of a family member in the house, they would think that any noise was the killer returning.

The Nepali CPSS was adapted from Foa EB, Johnson KM, Feeny NC, Treadwell KR: **The child PTSD Symptom Scale: a preliminary examination of its psychometric properties**. *Journal of Clinical Child Psychology* 2001, **30**(3):376-384.
